# How does morality work in the brain? A functional and structural perspective of moral behavior

**DOI:** 10.3389/fnint.2013.00065

**Published:** 2013-09-12

**Authors:** Leo Pascual, Paulo Rodrigues, David Gallardo-Pujol

**Affiliations:** ^1^Department of Personality, University of BarcelonaBarcelona, Spain; ^2^Mint Labs S.L.Barcelona, Spain; ^3^Institute for Brain, Cognition, and Behavior (IR3C), Universitat de BarcelonaBarcelona, Spain

**Keywords:** fMRI, morality, neuroscience, moral judgement, social brain, neuroimaging

## Abstract

Neural underpinnings of morality are not yet well understood. Researchers in moral neuroscience have tried to find specific structures and processes that shed light on how morality works. Here, we review the main brain areas that have been associated with morality at both structural and functional levels and speculate about how it can be studied. Orbital and ventromedial prefrontal cortices are implicated in emotionally-driven moral decisions, while dorsolateral prefrontal cortex appears to moderate its response. These competing processes may be mediated by the anterior cingulate cortex. Parietal and temporal structures play important roles in the attribution of others' beliefs and intentions. The insular cortex is engaged during empathic processes. Other regions seem to play a more complementary role in morality. Morality is supported not by a single brain circuitry or structure, but by several circuits overlapping with other complex processes. The identification of the core features of morality and moral-related processes is needed. Neuroscience can provide meaningful insights in order to delineate the boundaries of morality in conjunction with moral psychology.

“By four-thirty in the morning the priest was all cleaned up. I felt a lot better. I always did, after. Killing makes me feel good. (…) It's a sweet release, a necessary letting go of all the little hydraulic valves inside. (…) It has to be done the right way, at the right time, with the right partner—very complicated, but very necessary.”

Dexter, Darkly dreaming Dexter (Jeff Lindsay)

Can immoral behavior sometimes turn out to be moral? What mechanisms underlie morality? The above quotation is taken from a scene in the American TV series “Dexter.” Dexter is a respected employee at the Miami Metro Police Department, and a family man. However, at night Dexter doubles as a covert serial killer, applying his own moral code and murdering assassins whom the legal system has failed to condemn or catch. To what extent can a murder be considered necessary or even moral? Dexter's code includes clear examples of moral paradoxes that are not yet well understood. Does Dexter's brain work in the same way as the brains of other people? Researchers in moral neuroscience have tried to find domain-specific structures and processes that shed light on what morality is and where it is in the brain, if in fact it is there at all.

In this article, we focus on the history of the scientific study of neuroscience and the ways in which it has approached morality. We briefly review the main brain areas that have recently been associated with morality at both structural and functional levels and then discuss some of the implications of our review. We also speculate about how morality can be studied from the point of view of neuroscience. Here, we did a comprehensive review based on database search and references' search complemented with Neurosynth as a tool to conduct reverse and forward inferences (Yarkoni et al., 2011).

## What is morality?

Morality has traditionally been regarded as a code of values guiding the choices and actions that determine the purpose and the course of our lives (Rand, 1964). Recently, it has been operationalized as a code of conduct that, given specified conditions, would be put forward by all rational persons (Gert, 2012). From a scientific point of view, the studies by Kohlberg represented a milestone in the psychological study of morality (Kohlberg, 1963, 1984). Kohlberg considered moral reasoning to be a result of cognitive processes that may exist even in the absence of any kind of emotions. However, findings in evolutionary psychology (Trivers, 1971; Pinker, 1997) and primatology (Flack and de Waal, 2000) suggested that emotions played a key part in the origins of human morality (e.g., kin altruism, reciprocal altruism, revenge).

Today, there is a general consensus in psychology and philosophy in favor of the differentiation of moral processes into two different classes: (1) rational, effortful and explicit, and (2) emotional, quick and intuitive (De Neys and Glumicic, 2008). The controversy remains, though, in how they interact. Among current models of moral processes and how they relate to each other, three distinct theories outstand (Greene and Haidt, 2002; Moll and Schulkin, 2009). The “*social intuitionist theory*” (Haidt, 2001) links research on automaticity (Bargh and Chartrand, 1999) to recent findings in neuroscience and evolutionary psychology. The “*cognitive control and conflict theory*” (Greene et al., 2004) postulates that responses arising from emotion-related brain areas favor one outcome, while cognitive responses favor a different one (Kahneman and Frederick, 2007; McClure et al., 2007). According to the “*cognitive and emotional integration theory*,” behavioral choices cannot be split into cognitive vs. emotional. Complex contextual situations can make behavioral decisions exceptionally difficult (Gottfried, 1999; Moll et al., 2003).

## How can morality be studied scientifically?

A variety of methods for exploring morality have been developed, from moral vs. non-moral situations to moral dilemmas (Young and Dungan, 2012). Moral dilemmas are situations in which every possible course of action breaches some otherwise binding moral principle (Thomson, 1985). The two main distinctions between moral dilemmas and judgments that have traditionally been taken into account are: (1) personal dilemmas and judgments, as opposed to impersonal ones (Greene et al., 2004); (2) utilitarian moral judgments vs. non-utilitarian ones (Brink, 1986).

These distinctions have led to the development of a variety of paradigms. Probably the most famous ones are the trolley paradigm (Thomson, 1985) and the footbridge dilemma (Navarrete et al., 2012). In both the trolley problem and the footbridge dilemma, the choice is between saving five people at the expense of killing one person or letting five die and one survive (Hauser, 2006; Greene, 2007). However, the latter meets the criteria of a personal dilemma, while the former does not (for extensive reviews of similar moral dilemmas, see Greene et al., 2004; Koenigs et al., 2007; Decety et al., 2011; Pujol et al., 2011). Other tasks that bring morality under experimental scrutiny present visual sentences or pictures (Greene et al., 2001; Harenski and Hamaan, 2006), or scales and questionnaires that can be used to assess moral behavior from a clinical point of view (see Rush et al., 2008 for a review).

## The neuroanatomy of morality

**Figure 1:**
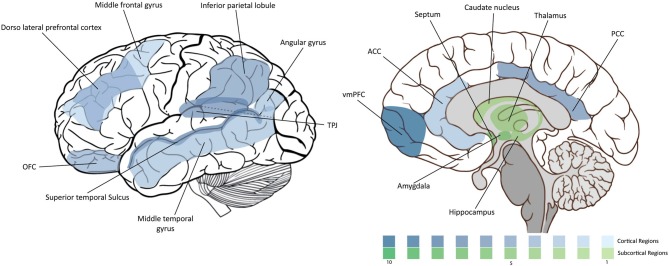
**Density of moral neuroscience studies**. The intensity of the color is proportional to the number of the citations of the corresponding region in the article.

Being a highly complex process, morality involves a highly complex neural circuitry. In this section we overview the main brain areas and circuitry that have been associated with it. The “moral brain” comprises a large functional network that includes several brain structures. At the same time, many of these structures overlap with other regions that control different behavioral processes. We will review them in the following order: (1) the frontal lobe, (2) the parietal lobe, (3) the temporal lobe and insula, and (4) the subcortical structures. The findings are summarized in Figure 1 and Table 1.

**Table 1:** **Studies that have addressed the morality problem from a neuroscientific viewpoint**.

	**VMPFC**	**OFC**	**DLPFC**	**ACC**	**MFG**	**IPR**	**TPJ**	**STS**	**A/MTG**	**AG**	**PCC**	**IC**	**HIP**	**AMG**	**THL**	**SPT**	**CN**
Partiot et al. (1995)					X												
Bush et al. (2000)				X													
Allison et al. (2000)						X		X									
Greene et al. (2001)	X							X		X							
O'Doherty et al. (2001)		X															
Frith (2001)				X	X												
Moll et al. (2001)									X								
Farrow et al. (2001)											X						
Moll et al. (2002)	X	X						X				X					
Greene and Haidt (2002)						X											
Wicker et al. (2003)												X					
Greene et al. (2004)			X			X		X	X		X	X		X			
de Quervain et al. (2004)																	X
Moll et al. (2005)	X											X					
Jackson et al. (2005)												X			X		
Harenski and Hamaan (2006)	X	X							X					X			
Prehn et al. (2008)								X									
Borg et al. (2006)										X							
Völlm et al. (2006)											X	X					
Mendez (2006)														X			
Moll et al. (2006)																X	
Kent and Kiehl (2006)																X	
Luo et al. (2006)																	X
Koenigs et al. (2007)	X																
Young and Koenigs (2007)	X		X	X													
Ciaramidaro et al. (2007)							X										
Tsetsenis et al. (2007)													X				
Prehn et al. (2008)	X		X														
Haushofer and Fehr (2008)			X														
Harenski et al. (2008)						X		X									
Young and Saxe (2008)							X										
Buckholtz et al. (2008)											X			X			
Hsu (2008)												X					
Harada et al. (2009)	X		X				X										
Krajbich et al. (2009)	X																
Greene and Paxton (2009)			X														
Fusar-Poli et al. (2009)													X				
Funk and Gazzaniga (2009)										X	X						
Cooper et al. (2010)	X																
Shenhav and Greene (2010)		X															
Young et al. (2010)							X										
Cooper et al. (2010)												X					
Blair (2010)														X			
Sommer et al. (2010)															X		
Cáceda et al. (2011)						X											
Young and Dungan (2011)							X										
Moor et al. (2011)							X	X									
Sestieri et al. (2011)											X						
Decety et al. (2011)												X		X	X		
Immordino-Yang and Singh (2011)												X	X				
Total citations	10	4	6	3	2	5	6	7	3	3	6	10	3	6	3	2	2

VMPFC, ventromedial prefrontal cortex; OFC, orbitofrontal cortex; DLPFC, dorsolateral prefrontal cortex; ACC, anterior cingulate cortex; MFG, medial frontal gyrus; IPR, inferior parietal region; TPJ, temoro-parietal junction; STS, superior temporal sulcus; A/MTG, anterior/middle temporal gyrus; AG, angular gyrus; PCC, posterior cingulate cortex; IC, insular cortex; HIP, hippocampus; AMG, amigdala; THL, thalamus; SPT, septum; CN, caudate nucleus.

### Frontal lobe

The ventromedial prefrontal cortex (VMPFC) is consistently engaged in moral judgement (Greene et al., 2001; Moll et al., 2002; Harenski and Hamaan, 2006; Koenigs et al., 2007; Prehn et al., 2008; Harada et al., 2009). VMPFC seems to play a crucial role in the mediation of the emotions engaged during moral processing (Young and Koenigs, 2007). Patients with VMPFC lesions are reported to be significantly more likely to endorse utilitarian responses to hard personal moral dilemmas (Koenigs et al., 2007) and have trouble representing the abstract consequences of their decisions (Krajbich et al., 2009). It is also involved in adherence to social norms and values (Moll et al., 2005) and in the integration of representations of others' intentions with their outcomes during social decision-making (Cooper et al., 2010). The left VMPFC shows higher activation in subjects with lower moral judgment competence when identifying norm violations (Prehn et al., 2008).

The orbitofrontal cortex (OFC) has been associated with morality, and has been implicated in the on-line representation of reward and punishment (O'Doherty et al., 2001; Shenhav and Greene, 2010). The right medial OFC was found to be activated during passive viewing of moral stimuli compared with non-moral stimuli (Harenski and Hamaan, 2006), while the activation of the left OFC has been related to processing of emotionally salient statements with moral value (Moll et al., 2002). Greene et al. (2004) speculated that the orbital and ventromedial prefrontal cortices seem to be involved in emotionally driven moral decisions, whereas the dorsolateral prefrontal cortex (DLPFC) competes with it, eventually mitigating its responses (Greene et al., 2004). The DLPFC is differentially activated when subjects emit a utilitarian response (Young and Koenigs, 2007). This area is involved in cognitive control and problem-solving (Greene et al., 2004). The DLPFC plays an important role during the judgment of responsibility for crimes and its punishment from a third-party perspective (Haushofer and Fehr, 2008), and also in the analysis of situations that demand rule-based knowledge (Prehn et al., 2008). Greene and Paxton (2009) related it to lying processes, and others have hypothesized that it may trigger an executive function used to combine predictions based on social norms with inferences about the intent to deceive (Harada et al., 2009).

The anterior cingulate cortex (ACC) is involved in error detection (Shackman et al., 2011). It is activated when subjects generate a utilitarian response (Young and Koenigs, 2007). The ACC, among others, has been implicated in theory of mind (ToM) and self-referential tasks (Frith, 2001), and it has been involved in moral conflict monitoring (Greene et al., 2004, p. 391). The medial frontal gyrus is another frontal region that seems to intervene in ToM, and also in other social functions relevant to moral judgment (Amodio and Frith, 2006) and in the integration of emotion into decision-making and planning (Partiot et al., 1995).

### Parietal lobe

The inferior parietal region is mainly associated with working memory and cognitive control, and so its recruitment during moral processing might be due to some cognitive engagement (Greene et al., 2004; Harenski et al., 2008; Cáceda et al., 2011). One of its functions, together with the posterior area of the superior temporal sulcus (STS) which we will review below, seems to be the perception and representation of social information that may be crucial for making inferences about others' beliefs and intentions (Allison et al., 2000) and the representation of personhood (Greene and Haidt, 2002).

The temporo-parietal junction (TPJ) plays a key role in moral intuition and in belief attribution during moral processing in others (Young and Saxe, 2008; Harada et al., 2009; Young et al., 2010; Moor et al., 2011; Young and Dungan, 2011). The TPJ, as well as the precuneus, is involved in encoding beliefs and integrating them with other relevant features of the action such as the outcome (Young and Saxe, 2008). The right TPJ and the precuneus are active when subjects process prior intentions, while the left TPJ is activated when a subset of social intentions is involved (Ciaramidaro et al., 2007) as well as lying (Harada et al., 2009). The disruption of the right TPJ activity affects the capacity to use mental states during moral judgment (Young et al., 2010). In the dictator game, activation in the TPJ is associated with punishment of the excluders through lower offers (Moor et al., 2011).

### Temporal lobe

Temporal lobe is one of the main neural regions activated during ToM tasks (Völlm et al., 2006; Ciaramidaro et al., 2007; Muller et al., 2010). Structural abnormalities within this area have even been related to psychopathy (Blair, 2010; Pujol et al., 2011).

One of the main temporal sub-regions involved in moral judgment is the superior temporal sulcus (STS) (Allison et al., 2000; Moll et al., 2002; Greene et al., 2004; Harenski et al., 2008). This structure has been understood as an initial site of social perception (Allison et al., 2000) and has been repeatedly associated with emotional processing and social cognition (Greene et al., 2004; Harenski et al., 2008). The STS has been described as indispensable for making inferences about others' beliefs and intentions (Allison et al., 2000). Increased activity of this area is also observed in personal dilemmas compared with other types (Greene et al., 2001). In the dictator game the STS has been found to be activated when subjects applied punishment to the excluders (Moor et al., 2011). The posterior STS shows greater activity during justice-based dilemmas than in care-based dilemmas (Harenski et al., 2008). Subjects with lower moral judgment competence showed greater activation in the left posterior STS when identifying norm violations (Prehn et al., 2008).

The anterior/middle temporal gyrus has been also related to moral judgment (Moll et al., 2001; Greene et al., 2004; Harenski and Hamaan, 2006). Angular gyrus engagement has been observed during the evaluation of personal moral dilemmas (Greene et al., 2001; Borg et al., 2006; Funk and Gazzaniga, 2009).

### Limbic lobe

The posterior cingulate cortex (PCC) is known to be involved in the processing of personal memory, self-awareness and emotionally salient stimuli (Sestieri et al., 2011). It is one of the brain regions that exhibit greater engagement in personal than in impersonal dilemmas (Funk and Gazzaniga, 2009). Its activation has been related to social ability (Greene et al., 2004), empathy (Völlm et al., 2006) and forgiveness (Farrow et al., 2001), and can predict the magnitude of the punishments applied in criminal scenarios (Buckholtz et al., 2008).

The insular cortex is also engaged in moral tasks (Moll et al., 2002; Greene et al., 2004). It exhibits greater activation in first-person and other-person experiences of disgust (Wicker et al., 2003). It is associated with emotional processing (Greene et al., 2004), empathic sadness in young subjects (Decety et al., 2011), detection and processing of uncertainty (Cooper et al., 2010) and perception of inequity (Hsu, 2008).

The anterior insular cortex is involved in visceral somatosensation, emotional feeling and regulation, and empathy (Immordino-Yang and Singh, 2011). This sub-region is activated during the experiencing of anger or indignation (Wicker et al., 2003; Moll et al., 2005), and when perceiving or assessing painful situations in others (Jackson et al., 2005). Its activation is also correlated with empathy scores (Völlm et al., 2006) and with unfair offers in a ‘ultimatum game’ (Hsu, 2008).

## Subcortical structures

The hippocampus is known to be a crucial region for the acquisition and retrieval of fear conditioning (Tsetsenis et al., 2007) and plays a facilitative role in inducing appropriate emotional reactions, in self-related processing during social emotions (Immordino-Yang and Singh, 2011) and in the processing of emotional facial expressions (Fusar-Poli et al., 2009).

The amygdala is a necessary structure for moral learning (Mendez, 2006). It is involved in the evaluation of moral judgments (Greene et al., 2004) and in empathic sadness during morally-salient scenarios (Decety et al., 2011). It can predict punishment magnitude in criminal scenarios (Buckholtz et al., 2008). Its dysfunction has been implicated in the affective deficits in psychopathy (Blair, 2010).

Rating empathic sadness, and perceiving and assessing painful situations has been associated with significant activation changes in the thalamus (Jackson et al., 2005; Decety et al., 2011). Bilateral thalamic activations are also observed when subjects are asked to choose between following a moral rule or a personal desire (Sommer et al., 2010).

The septum is activated while subjects make charitable contributions (Moll et al., 2006) and has been associated with psychopathy (Kent and Kiehl, 2006). Finally, the caudate nucleus is activated during altruistic punishment (de Quervain et al., 2004) and during the evaluation of morally salient stimuli (Luo et al., 2006).

## Discussion and conclusion

Moral neuroscience is an intricate and expanding field. This review summarizes the main scientific findings obtained to date. Morality is a set of complex emotional and cognitive processes that is reflected across many brain domains. Some of them are recurrently found to be indispensable in order to emit a moral judgment, but none of them is uniquely related to morality. The orbital and ventromedial prefrontal cortices are implicated in emotionally-driven moral decisions, whereas the dorsolateral prefrontal cortex seems to mitigate the salience of prepotent emotional responses. These competing processes may be monitored by the anterior cingulate cortex, which is also crucial for ToM. The TPJ and the STS play important roles in the attribution of others' beliefs and intentions. The insular cortex is engaged during empathic processes, and seems to be in charge of the evaluation of disgust and inequity. Other regions such as the posterior cingulate cortex, the anterior/middle temporal gyrus and the inferior parietal lobe seem to play a more complementary role in morality, being recruited in order to accomplish general cognitive processes engaged during the moral tasks proposed (e.g., working memory **or** cognitive control). On the other hand, regions like the amygdala seem to play an important role in the processing of emotions involved in moral judgment. Some of the emotions processed are more central to morality than others, but all emotions contribute to moral judgment given specific contextual situations.

The neural circuits of brain regions implicated in morality overlap with those that regulate other behavioral processes, suggesting that there is probably no undiscovered neural substrate that uniquely supports moral cognition. The most plausible option is that the “moral brain” does not exist *per se*: rather, moral processes require the engagement of specific structures of both the “emotional” and the “cognitive” brains, and the difference with respect to other cognitive and emotional processes may lie in the content of these processes, rather than in specific circuits. Some authors, though, have related morality to basic emotions such as *disgust* (Chapman et al., 2009). Further research is needed in order to uncover the relationships between basic emotions and morality, as well as basic cognition blocks such as attentional control (van Dillen et al., 2012).

Given that morality is a highly complex process influenced by many factors, future studies should take into account individual differences (e.g., personality, genetics, religiosity, cultural and socioeconomic level) in order to understand the variety of mechanisms that govern it. Genetic factors and environmental-dependent processes during developmental stages may strengthen specific neural circuits that process various moral dimensions (Gallardo-Pujol et al., submitted).

Another important constraint in moral research is the heterogeneity of the tasks used in different studies to assess morality, which makes the comparison of the different results extremely difficult. Moreover, some of the tasks proposed barely suggest actual daily moral situations and usually require abstract evaluation, a circumstance that may blur the results obtained. The inclusion of innovative techniques such as immersive virtual environments (Slater et al., 2006; Navarrete et al., 2012) adds apparent validity to moral dilemmas and may facilitate the generalization of results to real-life settings.

All in all, morality is supported not by a single brain circuitry or structure, but by a multiplicity of circuits that overlap with other general complex processes. One of the key issues that needs to be addressed is the identification of the core features of morality and moral-related processes. In this endeavor, neuroscience can provide meaningful insights in order to delineate the boundaries of morality in conjunction with moral psychology.

### Conflict of interest statement

The authors declare that the research was conducted in the absence of any commercial or financial relationships that could be construed as a potential conflict of interest.
